# The spatulated glass rod: low-tech instrument for high-impact ophthalmic diagnosis and treatment

**DOI:** 10.1007/s10792-025-03569-4

**Published:** 2025-05-28

**Authors:** Michelle Bai, Michele Y. Fu, Grace A. Borchert, Natalie S. Lee, Amitouj S. Sidhu, Ivy Jiang, Peter J. Tweedie, Susan C. Gaden, Ashish Agar, Ian C. Francis

**Affiliations:** 1 Sydney Eye Hospital, Sydney, Australia; 2 University of New South Wales, Sydney, Australia; 3 St Vincent’s Hospital, Sydney, Australia; 4 Nuffield Laboratory of Ophthalmology, Department of Clinical Neurosciences, Oxford University, Oxford, UK; 5 Royal North Shore Hospital, Sydney, Australia; 6 Concord Hospital, Sydney, Australia; 7 Department of Ophthalmology, Prince of Wales Hospital, Sydney, Australia; 8 Department of Ophthalmology, Northern Beaches Hospital, Sydney, Australia

**Keywords:** Glass rod, Corneal indentation, Double upper eyelid eversion, Head stabilisation technique, Ophthalmology

## Abstract

**Purpose:**

To describe the utility of the Spatulated Glass Rod (SGR), which is a low-tech but diagnostically and therapeutically high-impact instrument used in Ophthalmology for at least twelve clinical and surgical tasks.

**Methods:**

A literature review was conducted, examining the use of the glass rod both historically and in its applications in Ophthalmology. The MEDLINE database was searched for the terms ‘double eversion of upper eyelid’, ‘retained contact lens”, ‘spatulated glass rod’ and ‘spatulated glass rod in ophthalmology’.

**Results:**

Detailed descriptions are provided of the specific use of the SGR in twelve disorders, but particularly in the emergent management of acute angle closure using corneal indentation, and inspection of the upper fornix for elusive foreign bodies by double eversion of the upper lid. The authors’ Head Stabilisation Technique (HST) is also described as this facilitates safe head stabilisation by a novice assistant during the real-time application of the SGR.

**Conclusion:**

The SGR has proven to be an extremely useful instrument, with at least twelve different applications in both diagnostic and therapeutic contexts in Ophthalmology. Safe and effective use of the SGR is supported by the HST.

## Introduction

In recent decades, there have been numerous extraordinary high-tech developments that have revolutionised the discipline of Ophthalmology, and have collectively served to improve patient outcomes. These include diagnostic techniques such as optical coherence tomography, and therapeutic techniques including intravitreal injections of vascular endothelial growth factor inhibitors, Descemet membrane endothelial keratoplasty, stents for glaucoma, vitreoretinal surgery for macular holes, and endoscopic dacryocystorhinostomy.

These advanced and complex techniques distinctly contrast with the simplicity of the currently used Spatulated Glass Rod (SGR). While glass rods were first described in the first century AD, when used as stirring rods in Chemistry, it was not until the early twentieth century that glass rods were first utilised in Ophthalmology. In fact, in 1949, Thomassen referenced Ascher’s ‘glass rod phenomenon’ in assessing aqueous veins and blood flow in the diagnosis of glaucoma [1].

**Fig. 1:**
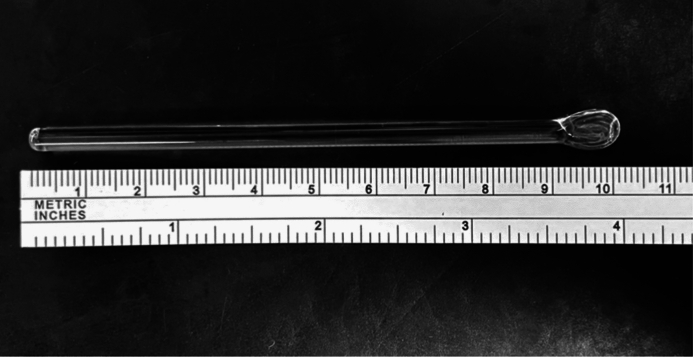
The spatulated glass rod

The SGR itself is an inexpensive and robust low-tech instrument which possesses a cylindrical glass handle, typically measuring 102 mm in length, and has a spatulated tip (Fig. 1). The shaft is 4.2 mm wide, with the spatulated tip measuring 7.9 mm by 2.4 mm (measured using the *FORTRADE® Surgical Callipers E1-7160, London, Englan*d). A package of 30 SGRs can be purchased from a medical supplier in Sydney, Australia, for AUD$240, representing an outlay of AUD$8 per unit. Ophthalmologists are thereby provided with a low-tech instrument by which they may perform, safely and effectively, at least twelve clinical and surgical tasks which effectively are clearly at the high-impact end of management for an individual patient.

## Methods

A literature review of the use of the glass rod, both historically and in its applications in Ophthalmology, was conducted utilising the University of New South Wales database access. The frequency and technique of double eversion (DE) of the upper eyelid in wider clinical practice were also examined. The search was conducted across MEDLINE from its year of inception in 1964 to the date of the search in September 2024, with the search terms ‘double eversion of upper eyelid’. The exclusion criteria discarded papers that did not mention DE. All articles retrieved were written in English. The authors’ literature review included case reports only if they explicitly described or included DE. Further MEDLINE search, involving the same publication period, was conducted with the search terms ‘retained contact lens”, ‘spatulated glass rod’ and ‘spatulated glass rod in ophthalmology’.

## Results

### Double eversion of upper eyelid

Using the search term ‘double eversion of upper eyelid’, 15 articles were retrieved, from 1979 to 2023, of which seven were included. Eight articles were excluded as six articles did not mention DE, and the full texts of two articles were unavailable.

Of the seven papers identified that mentioned DE, only four described their DE technique. Of those four, none reported that they utilised the glass rod.

Of significance, a simultaneous MEDLINE search for ‘retained contact lens’ returned 208 publications in total, 75 of which had been published in the previous eight years. This suggests that despite the relatively common incidence of foreign bodies being found in the upper fornix, and particularly contact lenses, DE is not frequently described, and possibly not utilised, in the evaluation and management of ‘lost’ upper fornix contact lenses.

### Spatulated glass rod

A MEDLINE search for ‘spatulated glass rod’ and ‘spatulated glass rod in ophthalmology’ was carried out and demonstrated no publications. Accordingly, the authors assembled their experiential data with the use of the SGR, and produced the documented applications of the SGR (Table 1), highlighting its importance in Ophthalmological practice [2–5]. While each of these applications is self-evident, they have all withstood the test of time in facilitating appropriate Ophthalmological diagnosis and therapy.

**Table 1:** Potential applications and uses of the spatulated glass rod (SGR) in ophthalmology

1. Facilitation of definitive management of acute angle closure by employing corneal indentation
2. Facilitation of single upper eyelid eversion using the non-spatulated end of the SGR
3. Facilitation of recognition and removal of elusive foreign bodies using the spatulated end of the SGR, such as ‘lost’ contact lenses or small foreign bodies, located on the supratarsal conjunctiva or in any of the four fornices, in combination with double eversion of the upper eyelid
4. Identification and removal of solid fragments of toxic substances, such as highly alkaline lime granules, from any fornix, under direct vision
5. Division of upper, lower, and temporal forniceal conjunctival shortening in patients with acute cicatrising conjunctivitis, such as Toxic Epidermal Necrolysis or Stevens-Johnson Syndrome
6. Assistance in the diagnosis and management of the giant fornix syndrome
7. Assistance with safe, controlled transconjunctival infiltration of local anaesthetic employing FLAT anaesthesia of the eyelid, by enabling direct visualisation of the conjunctival injection site
8. Indentation of the paracentral cornea to evaluate intraocular pressure when the lids are tightly apposed to the globe, in which situation applanation or rebound tonometry cannot be utilised. Examples include those of patients with tense orbits including acute surgical orbital haemorrhage and severe orbital cellulitis
9. Confirmation of a positive Seidel test using an SGR, achieved by compressing/releasing the possible site of leakage
10. Facilitation of controlled, transient anterior chamber decompression following cataract surgery in early-postoperative hypertensive eyes, employing localised SGR pressure directed anterior or posterior to the clear corneal incision
11. Evaluation of phthisis bulbi or other forms of hypotony because of globe distortion, when standard tonometry techniques are inadequate
12. Establishment of the diagnosis of a pseudo-pterygium

*FLAT* facilitated lid anaesthesia technique; *SGR* spatulated glass rod

### Head stabilisation technique

The Head Stabilisation Technique (HST) facilitates multiple procedures on the slit lamp. It is particularly useful when the clinician’s assistant is naïve to the importance of immobilising the patient’s head during any slit lamp procedure. It permits numerous common slit lamp procedures such as corneal foreign body and suture removal to be done safely. In this study, it allows for the safe and effective corneal indentation (CI), and identification of forniceal foreign bodies by employing the DE technique.

The naïve assistant, standing on one side of the patient seated at the slit lamp, uses his or her ipsilateral hand to push the patient’s occiput forwards onto the forehead rest and chin rest of the slit lamp. The assistant’s contralateral hand is placed onto the forehead rest as well as the patient’s forehead, ensuring that the patient’s forehead is in constant contact with the forehead rest, stabilising them there. The patient is encouraged to maintain steady breathing through the nose, with the mouth closed and the teeth apposed. Patients are exhorted not to breath-hold, as this tends to make them look up, become restless, and move posteriorly off the slit lamp.

The authors have shown, in over four years of carrying out HST, that this technique is easily learned by naïve assistants, and the clinician is certain that the patient’s head is securely immobilised on the slit lamp.

### Technique of corneal indentation

**Fig. 2:**
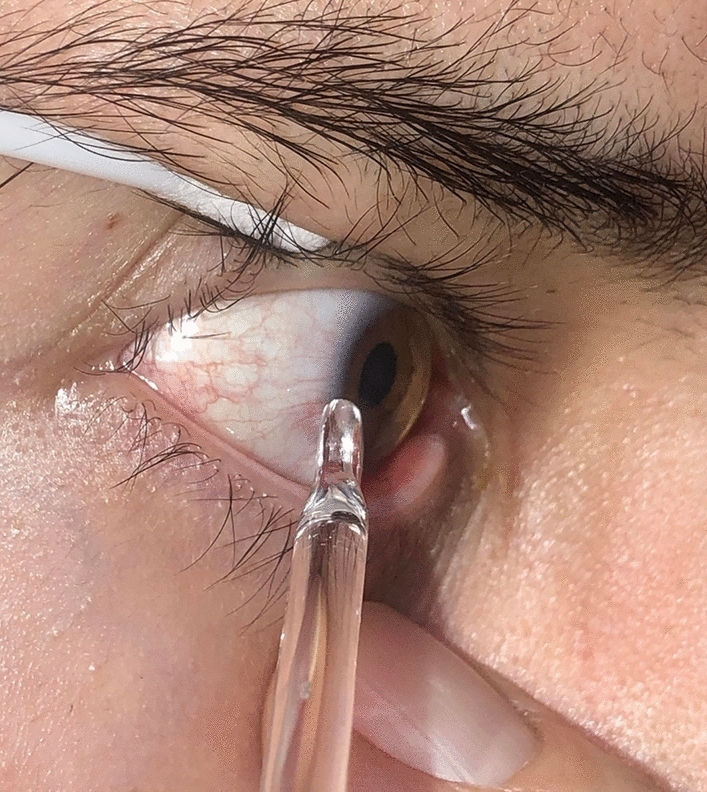
Corneal indentation using the spatulated end of the spatulated glass rod (note that the spatulated end of the SGR is seen side-on here)

A recently well-documented, published, and now increasingly widely accepted technique in the emergent management of acute angle closure (AAC), is definitive CI [4, 5]. Following instillation of topical oxybuprocaine eye drops, with the patient seated and immobilised on the slit lamp and looking straight ahead, the clinician applies anteroposterior compression on the limbus/cornea with the SGR, for repeated cycles of 30 seconds on and 30 seconds off (Fig. 2). Generally, fewer than ten cycles are required to control AAC, normalising the intraocular pressure. This results in almost immediate resolution of corneal oedema, permitting safe YAG laser peripheral iridotomy. If necessary, cataract and implant surgery can be carried out later, as this definitively deepens the anterior chamber and prevents AAC [6].

### Technique of double eversion of the upper eyelid

A detailed step-by-step description follows, of DE of the upper eyelid using the SGR. DE should be performed gently, to avoid patient discomfort, but also carefully, to avoid missing a small and decolourised upper fornix foreign body [7]. The DE technique enables visualisation of the superior conjunctival fornix, and has a substantial track record of success in the authors’ hands. If DE is performed well, as described, patient distress is obviated, and the risks of inadequate diagnosis, subsequent incorrect treatment, and possible complications are minimised. DE is optimally facilitated using the SGR, but, in contrast to single eversion of the upper eyelid, requires the instillation of topical anaesthetic.

Following administration of topical anaesthetic, the patient’s head is immobilised on the slit lamp. Assistance from an experienced assistant ensures safe and effective head stabilisation while DE is carried out. Alternatively, if only naïve assistants are present, the authors’ HST, described above, may be employed.

The patient is then asked to look down ‘with both eyes open’ towards the slit lamp table. This downgaze minimises the contraction of the orbicularis oculi, relaxes the levator aponeurosis and Muller’s muscle, thereby loosening the superior component of the orbital diaphragm, facilitating easier DE. Furthermore, with both eyes open while looking down, the usually upgoing Bell’s phenomenon is obviated, as it could potentially result in re-inversion of the upper lid during DE [8].

To achieve single eversion of the upper lid, topical anaesthesia is not usually required. In this technique, again with the patient in downgaze, the clinician’s non-dominant index finger and thumb grasp the patient’s upper eyelid eyelashes, and the non-spatulated end of the glass rod is placed in the upper eyelid crease. The upper eyelid is then everted using the eyelid crease/superior tarsal margin as a fulcrum. The clinician’s thumb and forefinger maintain their grip on the patient’s eyelashes, securing the patient’s singly-everted upper lid in a stable everted position, and allowing detailed inspection of the tarsoconjunctiva.

**Fig. 3:**
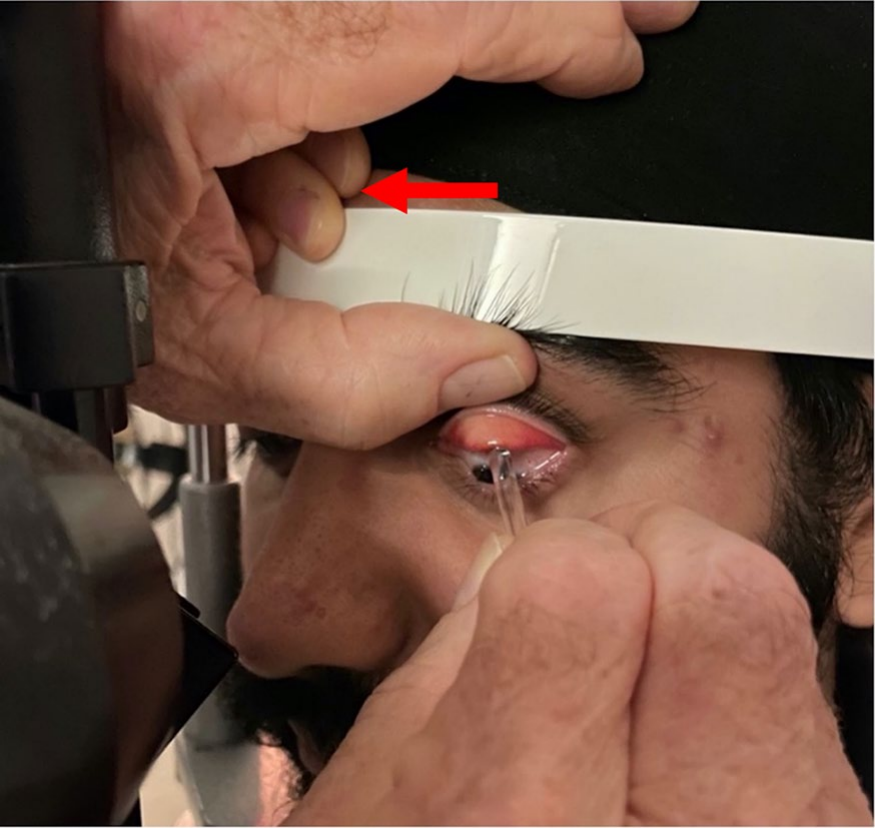
The spatulated glass rod is in place here prior to further eversion of the upper lid in order to inspect the fornix directly. Note that the clinician’s thumb is stabilising the patient’s upper lid. Note also that the assistant’s ipsilateral left hand (arrow) is ensuring that the patient’s forehead is firmly secured against the forehead rest of the slit lamp

For DE, topical anaesthesia is required. With the lid singly everted, and the patient’s head stabilised on the slit lamp, the clinician rotates the glass rod along its length and inserts the spatulated tip of the glass rod between the patient’s topically anaesthetised superior bulbar conjunctiva and the supratarsal conjunctiva. This is initially performed in the visual midline (Fig. 3). As the spatulated tip of the SGR moves superiorly, the patient’s upper eyelid is gently lifted away from the globe by pressure from the shaft of the SGR. For convenience, the SGR is usually moved from temporal to nasal in the right eye, and nasal to temporal in the left eye, allowing constant inspection on the slit lamp of the now-exposed upper fornix along its whole length. This permits direct inspection of the patient’s supratarsal conjunctiva, superior bulbar conjunctiva, and superior conjunctival fornix.

**Fig. 4:**
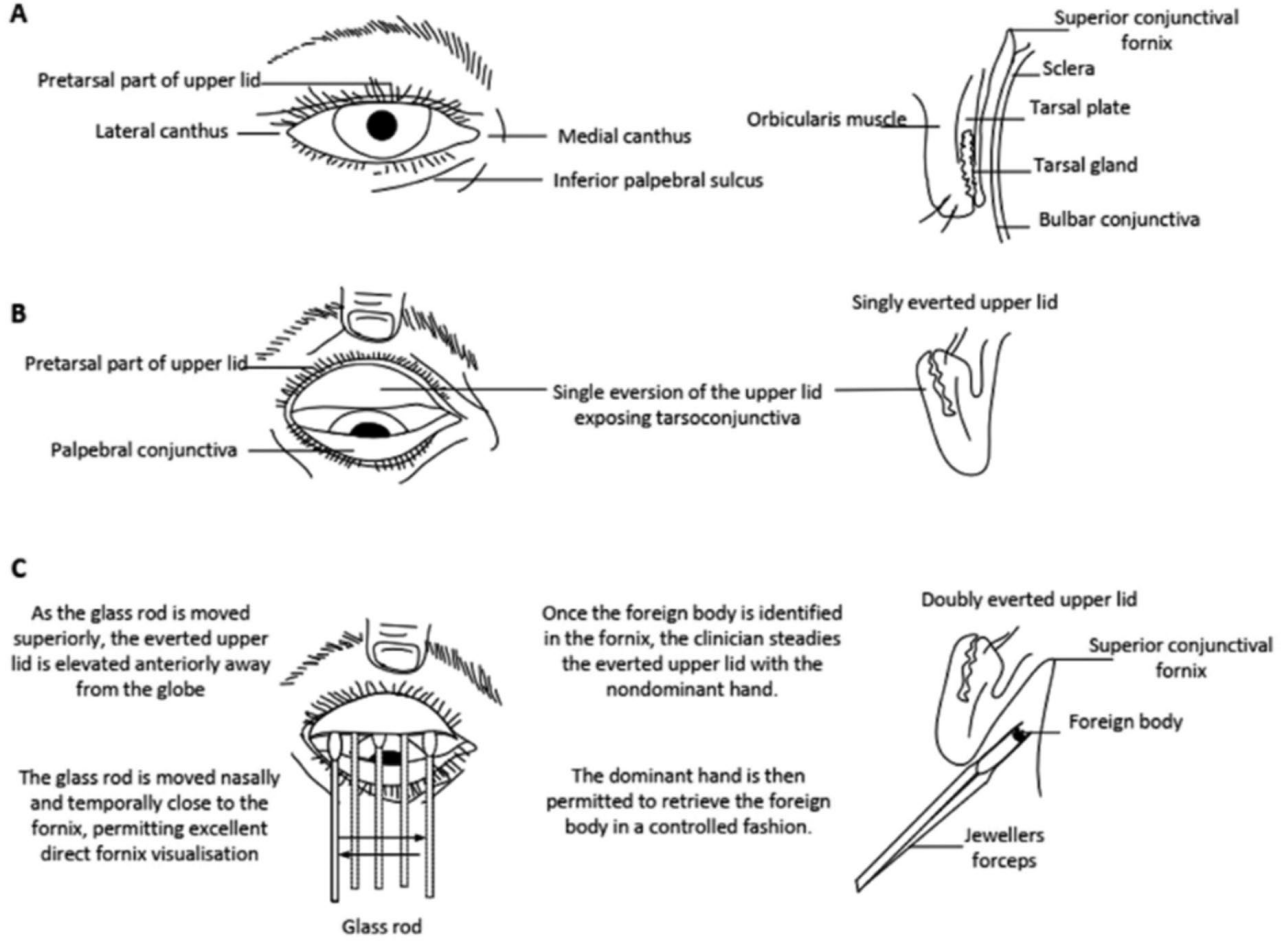
Original schematic of double eversion of the upper eyelid using the spatulated glass rod

The DE technique is demonstrated diagrammatically in Fig. 4. Again, the clinician moves the SGR in order to enable continuous visualisation of the superior fornix. This controlled visualisation of the whole length of the upper fornix enables immediate identification and removal of any foreign body.

The clinician, keeping the patient’s double-everted upper lid in position with the SGR, identifies the foreign body. While maintaining the foreign body in view, the clinician is handed the required forceps by the assistant. The foreign body is subsequently removed under direct vision with the benefit of slit lamp magnification and illumination. The maintained stabilisation of the patient’s head with the HST (when utilised) ensures safety when the forceps are used to retrieve the foreign body. This also minimises any possible patient discomfort. The forceps are removed from the field by handing them back to the assistant. The SGR is subsequently and immediately removed from the upper fornix.

Importantly, when the clinician releases the upper lid from its now singly-everted state, potential abrasion of the cornea by upper or lower eyelid lashes, when the lid re-inverts, is prevented by asking the patient to maintain downward gaze. The clinician simultaneously distracts the lower lid inferiorly with the index finger and thumb, to avoid abrasion by the lower lid lashes of the superior tarsoconjunctiva. This of course is more important in re-inverting a singly everted upper lid, where no topical anaesthesia is used.

## Discussion

The authors were impressed to read a 2012 study by Watanabe et al., out of both Adelaide, Australia, and Kyoto, Japan, in which ‘lost’ contact lenses were found in multiple conjunctival locations [9]. Watanabe et al. emphasised the importance of performing DE, which was often found to be the only means of visualising elusive foreign bodies in the upper fornix. They also reported 48 cases of retained contact lenses, of which 17 (35%) required DE for identification and management. In eight cases, contact lenses were discovered by single eversion. However, in 20 of their cases, it was unclear how the contact lenses were identified. Interestingly, in three cases, lid eversion of any form was not clinically necessary.

Therefore, when the presence of an upper fornix, inferior fornix, or lateral fornix foreign body is suspected, and single upper lid eversion fails to disclose the foreign body, DE with a SGR should be performed. Such an approach ensures that the foreign body can be directly visualised rather than being overlooked. It also prevents the foreign body from being forced subconjunctivally, or pushed further along the fornix. This approach also avoids ‘blind sweeping’ of the fornices, such as with a dampened cotton bud.

A 2020 study by Arshad et al. described a case in which a contact lens-wearing patient presented with a five-month history of discharge from one eye [10]. While Arshad et al. performed DE in an attempt to locate a lost contact lens, this was carried out with ‘blind sweeping’ of the fornices. The instrument used for this procedure was not identified. Furthermore, it was not clear whether the authors used a specific HST to achieve stability of the patient’s head during the procedure, nor whether topical anaesthesia was utilised. Whilst these authors should be commended for their utilisation of DE in this case, it is important to note that ‘blind sweeping’ of the upper fornix may have unfavourable outcomes, as outlined above. Not only may the forniceal foreign body be forced into the subconjunctival tissue of the fornix, but it may also migrate subcutaneously as a lump visible on the anterior upper eyelid [11]. If the forniceal tissue is already infected, or becomes infected, there may be a risk of orbital cellulitis. Further, using a cotton bud may liberate its own fibres into the fornix, thereby potentially exacerbating the patient’s original symptomatology and pathology.

A 2017 study by Ho and Matthews reported performing DE to retrieve a foreign body from a patient with bilateral dry eye symptoms [12]. These authors described the use of a cotton bud to sweep the fornices, but not under direct vision. In addition to risking the abovementioned complications, this ‘blind sweeping’ may result in direct trauma to the conjunctiva, the cornea, and possibly even the globe. Furthermore, as mentioned above, the cotton bud may, even when dampened, deposit its cotton fibres into the fornix.

Similarly, in 2013, Agarwal et al. described what could be regarded as an equally, but potentially suboptimal, method of DE [13]. They performed DE using a cotton bud in a patient with a retained soft contact lens. A similar study by Zola et al. described a patient with a four-year history of unilateral ptosis, irritation, and discharge [7]. These authors successfully removed a retained hard contact lens by sweeping the fornices without direct visualisation, but did not describe the instrument used. A 1986 study by Burns and Cahill also reported using a cotton bud to sweep the fornices without direct tissue visualisation, in a patient with a retained hard contact lens [14].

A 2009 study by Morris and Dolman reported the utilisation of a Desmarres retractor to perform DE and visualise a circular lesion consistent with an embedded hard contact lens [15]. However, their precise technique was not described. Further, it is notable that even a small Desmarres retractor is relatively invasive and may produce significant pain in attempting to visualise the upper fornix because of the associated stretching of eyelid tissue.

The 1996 publication by Goethals et al. described a 6-year-old patient with a foreign body seen only on DE of the upper eyelid [16]. The report suggested that DE was achieved by utilising a pair of forceps. This was presumably performed under general anaesthesia, and in the operating room, although the details were not provided.

The 2017 publication by Bhalerao et al. described a 63-year-old patient with a 1.5-month history of irritation and congestion in his right eye [17]. DE of the right upper eyelid revealed a honeybee sting of approximately 2 mm in size in the subconjunctival space. Without the benefit of slit lamp magnification and illumination, the honeybee sting might not have been visible to the naked eye. Using DE, the sting was safely and rapidly removed with forceps under direct vision.

A 2018 publication by Karim et al. described a case in which an elderly patient with giant fornix syndrome (GFS) was managed with regular ‘blind sweeping’ of the fornices with cotton buds [18]. Although GFS may ultimately necessitate conjunctival surgery [19], temporisation of the condition in elderly people can be achieved by removing foreign and infected forniceal material under direct vision [20]. On the other hand, Vahdani et al. were able to manage giant fornix syndrome with topical povidone-iodine and dexamethasone eye drops [21]. Intuitively, cotton buds should probably be avoided in the management of GFS due to their potential to liberate fibres into the fornix. Indeed, surgical spears may provide a safer method of cleaning the fornices. Therefore, managing GFS with DE, facilitated by the SGR, should render this a safer and more effective intervention.

The abovementioned publications, along with the current report, emphasise the value of DE of the upper eyelid. The authors consider these specific steps, described in the results, should allow DE to be performed in a painless, straightforward and safe fashion. Thus the SGR appears to be the ideal instrument for carrying out DE in Ophthalmology.

### Clinical case scenario

A 54-year-old man presented with a four-month history of intermittent irritation of his right eye on waking. He had been assessed by two optometrists and one consultant ophthalmologist without a diagnosis. The patient reported that when the irritation was present, he would rub his affected right eye, with symptom relief. Examination revealed only mild right conjunctival injection. There was no evidence of presumed nocturnal lagophthalmos [8]. Fluorescein staining demonstrated no abnormality, and upper and lower ipsilateral single eyelid eversion were normal.

DE was discussed with the patient and carried out. A tiny hair-like structure, covered in mucus, was retrieved from the upper fornix, slightly nasal to the midline. Following the procedure, the patient’s symptomatology resolved entirely.

## Conclusion

The SGR is a low-tech, inexpensive, reliable, sterilisable, reusable, robust, and easily accessible instrument. The authors have outlined twelve applications of the SGR in both diagnostic and therapeutic contexts, which may be helpful to ophthalmologists and others at the coalface. These applications represent a high-impact means of successfully managing a wide range Ophthalmological disorders. Utilisation of the SGR comes to the fore in the emergent management of AAC using CI. It also allows adequate evaluation of a patient’s upper fornices using DE, and facilitates the safe and efficacious removal of any foreign body or solid contaminant, most pertinently ‘lost’ contact lenses. It is reassuring that the multiple uses of the SGR have successfully withstood the test of time.

## Data Availability

No datasets were generated or analysed during the current study.
